# Supplementary material for: Blood flow-induced Notch activation and endothelial migration enable vascular remodeling in zebrafish embryos
Source: Nat Commun. 2018 Dec 14;9:5314. doi: 10.1038/s41467-018-07732-7 (PMC6294260; doi:10.1038/s41467-018-07732-7)
Supplement: Supplementary file 12 — Supplementary information [file 41467_2018_7732_MOESM12_ESM.pdf]

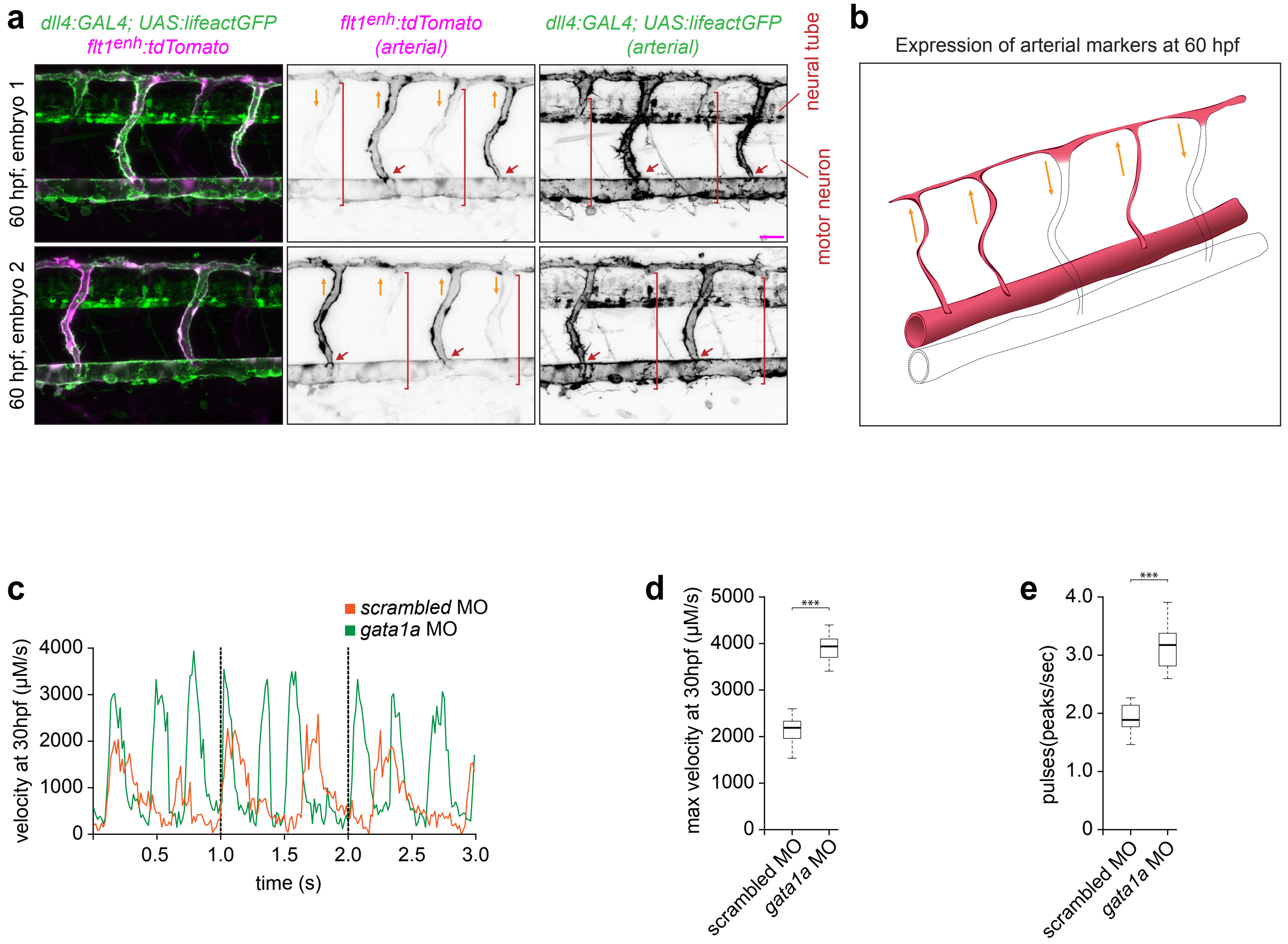

Supplementary Figure 1: Imaging of arterial ECs in zebrafish embryo.

a) *Tg(flt1^{enh}:tdTomato)* and *Tg(dll4:GAL4; UAS:lifeactGFP)* transgenic lines that label arterial ECs. Lateral images of zebrafish embryos at 60 hpf with anterior side facing left. *Orange arrows* indicate the direction of blood flow, *red arrows* point to arterial ISVs and *brackets* highlight regions of venous ISVs without arterial ECs. Note that, in addition to arteries, tdTomato is also weakly expressed in veins, whereas lifeactGFP is not detectable in veins but expressed in the neural tube and motor neurons. Scale bar is 25 µm. Images are representative from at least 3 independent experiments.

b) Schematic of the spatial expression of arterial markers.

c-e) Blood velocity in the DA embryos with and without erythrocytes. **c**) Line graph of velocity as a function of time. **d**) Box plot of maximal velocity. **e**) Box plot of the pulses.

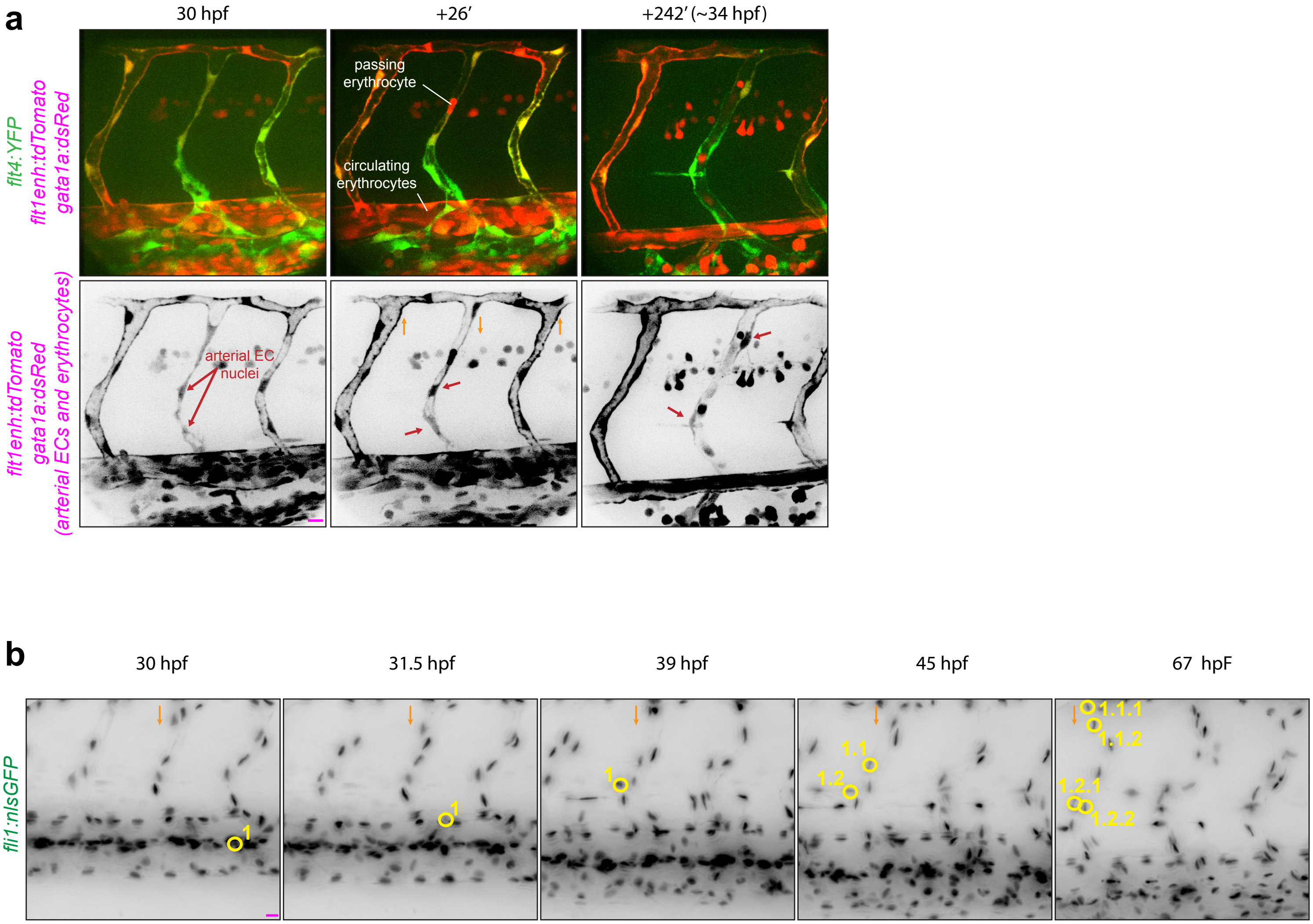

Supplementary Figure 2: Displacement of arterial ECs by venous ECs in venous ISVs.
All images are representative from at least 3 independent experiments. Lateral images of zebrafish embryos with anterior side facing left. *Orange arrows* indicate the direction of blood flow through the ISVs. Scale bars are 25 µm.
a) Stills from Supplementary Movie 1. Venous ECs are labelled with mCitrine, arterial ECs are labelled with mCitrine and tdTomato, and erythrocytes are labelled with dsRed.
b) Stills from Supplementary Movie 3. Proliferation of venous ECs in a venous ISV.

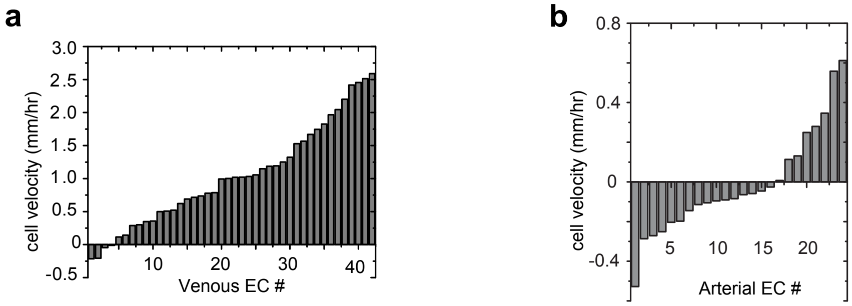

Supplementary Figure 3: Blood flow promotes EC migration in veins but not in arteries.
Histograms of velocities of venous (**a**) and arterial (**b**) ECs. Velocities are averaged over the course of experiment and ordered by their values. Velocity is considered positive, if the cell migrates upstream.

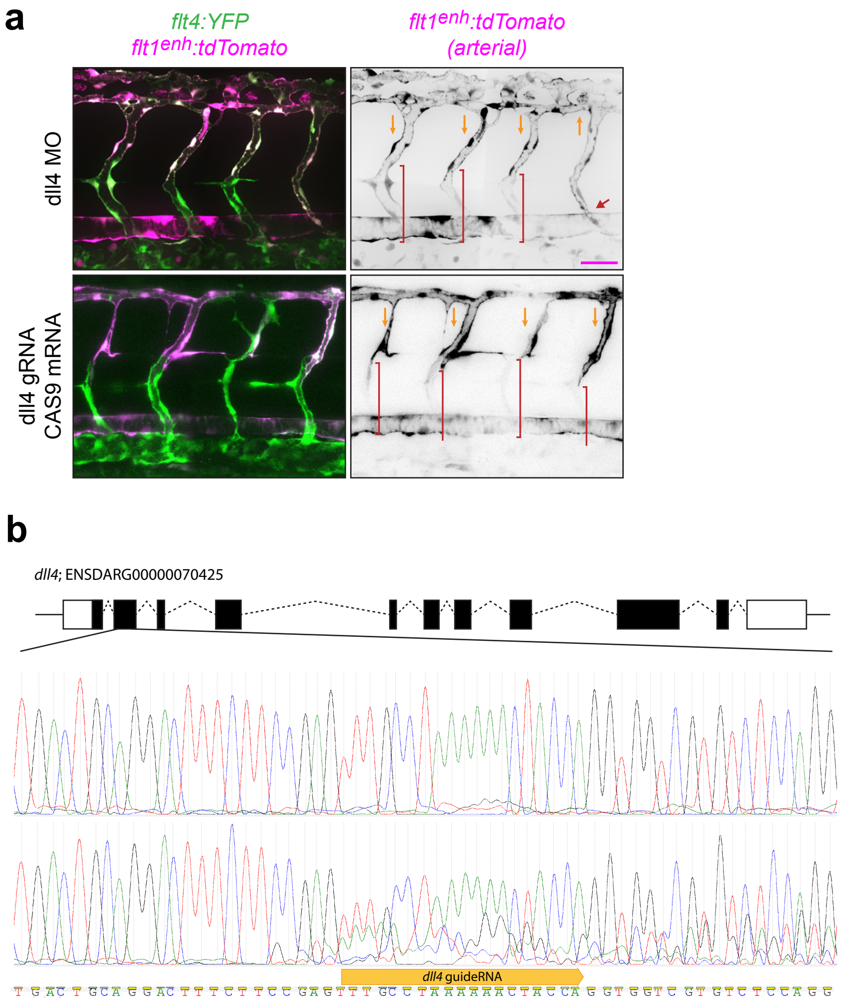

Supplementary Figure 4: Arterial flow protects ISVs from transforming into veins by activating Notch.
a) Lateral images of zebrafish embryos at 60 hpf with anterior side facing left. *Red arrows* point to arterial ISVs and *red brackets* highlight regions of venous ISVs without arterial ECs. *Orange arrows* indicate the direction of blood flow. Scale bars is 25 µm. Abrogation of *dll4* by MO or CRISPR-CAS9. Venous ECs are labelled with mCitrine and arterial ECs are labelled with mCitrine and tdTomato. All images are representative from at least 3 independent experiments.
b) CRISPR-CAS9 targeted location and representative sequencing plot of an injected embryo.
