# Supplementary material for: Blood flow-induced Notch activation and endothelial migration enable vascular remodeling in zebrafish embryos
Source: Nat Commun. 2018 Dec 14;9:5314. doi: 10.1038/s41467-018-07732-7 (PMC6294260; doi:10.1038/s41467-018-07732-7)
Supplement: Supplementary file 14 — Description of Additional Supplementary Files [file 41467_2018_7732_MOESM14_ESM.pdf]

Supplementary Movie Legends

Supplementary Movie 1. **Upstream migration of arterial ECs in a venous ISV.** Time-lapse imaging of *Tg(flt4:mCitrine; flt1^{enh}:tdTomato; gata1a:dsRed)* in which venous ECs are labelled with mCitrine (*green*), arterial ECs are labelled with mCitrine and tdTomato, and erythrocytes are labelled with dsRed (*red*). *Orange arrows* depict first erythrocyte passing through venous ISVs. *Green arrow* depicts arterial ECs migrating upstream in a venous ISV. *Red arrow* points to arterial ISVs.

Supplementary Movie 2. **Displacement of arterial ECs by venous ECs in a venous ISV.** Time-lapse imaging of *Tg(dll4:GAL4; UAS:lifeactGFP; fli1a:lifeactCherry)* in which venous ECs express lifeactCherry (*magenta*) and arterial ECs express lifeactCherry and lifeactGFP (*green*). *Red arrow* points to an arterial EC migrating dorsally in a venous ISV.

Supplementary Movie 3. **Migration and proliferation of venous ECs in a venous ISV.** Time-lapse imaging and cell tracing in *Tg(kdl:H2B-Cherry)*, in which all ECs express nuclear-localized mCherry (*grey*).

Supplementary Movie 4. **Arterial ECs do not migrate in an arterial ISV.** Time-lapse imaging of *Tg(dll4:GAL4; UAS:lifeactGFP; fli1a:lifeactCherry)* in which venous ECs express lifeactCherry (*magenta*) and arterial ECs express lifeactCherry and lifeactGFP (*green*). *Red arrow* points to an arterial EC in an arterial ISV.

Supplementary Movie 5. **Flow-induced changes in the planar polarization and migration of HUVECs in the microfluidic model.** Positions of MTOC and nuclei in individual HUVECs were monitored for 300 min after their exposure to flow with a shear stress τ = 7.2 dyn/cm^2. The direction of flow is from left to right. *Left panel* shows fluorescence images (with an inverted grayscale) of a fragment of a microchannel with ECs expressing GFP-α-tubulin. The arrows show the direction of cell polarization and their color indicates the polarization angle, β, with red corresponding to 90° (polarization against the flow) and blue corresponding to β = -90° (polarization along the flow). *Right panel* shows phase contrast images of the same area. The arrows show the direction of cell migration and their color represents the angle between the directions of migration and flow, β^*, with red corresponding to 90° (migration against the flow) and blue corresponding to β^* = -90° (migration along the flow). The dials in the top left corners show color-coded polarization angle, β (*left panel*), and migration angle, β^* (*right panel*) averaged over all cells in the field of view. Images were acquired every 10 min. Frame rate is 3 per sec (1 sec = 30 min). Scale bar is 30 μm.

Supplementary Movie 6. **Migration of HUVECs exposed to low and high shear stress in the microfluidic model.** Phase-contrast images of HUVECs exposed to a shear stress of 0.23 dyn/cm^2 (*upper panel*) and 14.5 (*lower panel*) acquired every 10 min. Direction of the flow was from left to right. Frame rate is 6 per sec. Scale bar is 50 μm.

Supplementary Movie 7. **Blood flow in arterial and venous ISVs.** Time-lapse imaging of *Tg(gata1:dsRed)*, in which all erythrocytes express dsRed (*grey*).

Supplementary Movie 8. **Blood flow in the dorsal aorta and posterior cardinal vein.** Time-lapse imaging of *Tg(gata1:dsRed)*, in which all erythrocytes express dsRed (*grey*).

Supplementary Movie 9. **Expression of *Tp1:d2GFP* during remodeling of the ISVs.** Time-lapse imaging of *Tg(Tp1:d2GFP; fli1a:lifeactCherry)* which expresses a de-stabilized GFP (*green*) under the control of 12xCSL Notch responsive elements. All ECs are marked by lifeactCherry (*magenta*). *Red arrows* point to arterial ISVs. *Green arrow* depicts a venous sprout that did not anastomose with an arterial ISV with high Notch activity.

Supplementary Movie 10. **Expression of *Tp1:d2GFP* during remodeling of the ISVs.** Time-lapse imaging of *Tg(Tp1:d2GFP; fli1a:lifeactCherry)* which expresses a de-stabilized GFP (*green*) under the control of 12xCSL Notch responsive elements. All ECs are marked by lifeactCherry (*magenta*). *Red arrows* point to arterial ISVs. *Green arrows* depict venous sprouts. The sprout on the *left* anastomoses with an ISV. *Orange arrow* highlights the formation of a lumen in the sprout on the right. This sprout detaches from an ISV aft er d2GFP becomes expressed. *Blue arrow* points to a venous EC migrating from the sprout into this ISV.

Supplementary Movie 11. **Expression of *Tp1:d2GFP* during remodeling of the ISVs.** Time-lapse imaging of *Tg(Tp1:d2GFP; fli1a:lifeactCherry)* which expresses a de-stabilized GFP (*green*) under the control of 12xCSL Notch responsive elements. All ECs express lifeactCherry (*magenta*). *Red arrows* point to arterial ISVs. *Green arrows* point to ECs expressing d2GFP in venous ISV.
